# Thermus javaensis sp. nov., a novel thermophilic bacterium isolated from litter of a geyser in Cisolok, West Java, Indonesia

**DOI:** 10.1099/ijsem.0.007136

**Published:** 2026-05-04

**Authors:** Fitria Ningsih, Mazytha Kinanti Rachmania, Dhian Chitra Ayu Fitria Sari, Yasunori Ichihashi, Song-Gun Kim, Shuhei Yabe, Wellyzar Sjamsuridzal

**Affiliations:** 1 Department of Biology, Faculty of Mathematics and Natural Sciences, Universitas Indonesia, Kampus UI Depok 16424, Indonesia; 2 Center of Excellence for Indigenous Biological Resources-Genome Studies, Faculty of Mathematics and Natural Sciences, Universitas Indonesia, Kampus UI Depok 16424, Indonesia; 3 RIKEN Center for Sustainable Resource Science, 3-1-1 Koyadai, Tsukuba, Ibaraki 305-0074, Japan; 4 Biological Resource Center/Korean Collection for Type Cultures (KCTC), Korea Research Institute of Bioscience and Biotechnology, Jeongeup, Jeonbuk 56212, Republic of Korea; 5 Department of Microbial Resources, Graduate School of Agricultural Science, Faculty of Agriculture, Tohoku University, 468-1 Aramaki Aza Aoba, Aoba-ku, Sendai, Japan; 6 Hazaka Plant Research Center, Kennan Eisei Kogyo Co.Ltd., 44 Inariyama, Ashitate, Shibata-gun, Miyagi 989-1311, Japan

**Keywords:** Cisolok Geyser, *Deinococci*, litter, thermophilic bacterium, *Thermus javaensis *sp. nov.

## Abstract

A thermophilic, yellow-pigmented bacterial strain, designated LT1-2-5^T^, was isolated from litter in the Cisolok Geyser, West Java, Indonesia. This strain is Gram-stain-negative, aerobic and non-sporulating, with rod-shaped cells and short filaments. Phylogenetic analyses based on 16S rRNA gene sequences (1,446 bp) placed strain LT1-2-5^T^ within the genus *Thermus*, showing highest similarities to *Thermus thalpophilus* SYSU G00506^T^ (98.54%) and *Thermus brockianus* YS038^T^ (98.34%). The strain exhibited optimal growth at 60–65 °C, pH 7.0–8.0 and ≤2.0% (w/v) sodium chloride (NaCl). Major cellular fatty acids (>10%) were iso-C_17:0_, iso-C_15:0_, anteiso-C_15:0_ and anteiso-C_17:0_. The polar lipids detected were phosphatidylethanolamine, phosphatidylglycerol, diphosphatidylglycerol and unidentified glycolipids. The major menaquinone was menaquinone-8. The genome size of strain LT1-2-5^T^ was 2.41 Mbp, and the DNA G+C content was 66.8 mol%. The average nucleotide identity and digital DNA–DNA hybridization values between strain LT1-2-5^T^ and *T. thalpophilus* SYSU G00506^T^ were 91.46% and 42.9%, respectively, both of which are below the established species cut-off value. Polyphasic taxonomy, including phylogenetic, genomic, phenotypic and chemotaxonomic analyses, confirmed the status of strain LT1-2-5^T^ as a novel species of *Thermus*, for which the name *Thermus javaensis* sp. nov. is proposed. The type strain is LT1-2-5^T^ (=UICC B-84^T^=CGMCC 1.61921^T^=KCTC 102310^T^).

## Introduction

The genus *Thermus*, first described by Brock and Freeze in 1969 [1], belongs to the family *Thermaceae* as proposed by da Costa and Rainey [2]. The members of this genus are primarily isolated from geothermal environments [1,8]. Since the description of the type species, *Thermus aquaticus* [1], the genus has expanded to include 25 validly published species according to the List of Prokaryotic Names with Standing in Nomenclature (LPSN) [9] data (https://lpsn.dsmz.de/genus/thermus), each contributing to our understanding of high-temperature microbial adaptation and diversification. This genus has become a focal point of microbiological research due to its ecological resilience and biotechnological potential [10,14].

Taxonomically, members of the genus *Thermus* are Gram-stain-negative, aerobic, heterotrophic, non-sporulating, rod-shaped bacteria that typically form yellow-pigmented colonies [10,12, 13, 15]. *Thermus* species can grow between 37 and 83 °C, with an optimum temperature of 65–70 °C. Key chemotaxonomic markers include menaquinone-8 (MK-8) as the major respiratory quinone, a prevalence of branched-chain fatty acids (e.g. iso-C_15:0_ and iso-C_17:0_) and a polar lipid profile dominated by glycolipids, phospholipids and aminophospholipids. Their genomic G+C content (mol%) is characteristically high, ranging from 61.7 to 71.3 mol% [1,6,13, 16].

While well-studied in regions like North America and Iceland [1,3], the geothermal ecosystems of Southeast Asia remain underexplored for novel *Thermus* diversity. The Cisolok Geyser in Indonesia, with its unique organic litter-rich environment, represents a promising source for discovering new thermophilic species [23,24].

In this study, we describe a new bacterial strain, LT1-2-5^T^, isolated from litter at the Cisolok Geyser, West Java, Indonesia. Through a polyphasic taxonomic approach, including 16S rRNA gene phylogeny, whole-genome comparisons [average nucleotide identity (ANI)/digital DNA–DNA hybridization (dDDH)] and chemotaxonomic profiling, we propose the name *Thermus javaensis* sp. nov. for this strain. This discovery expands the known diversity of the genus and underscores the taxonomic significance of Indonesian geothermal habitats.

### Isolation

Strain LT1-2-5^T^ was isolated from litter collected at the Cisolok Geyser, Sukabumi, West Java, Indonesia (6° 56' 00.4" S 106° 27' 13.1" E) on 26 September 2015. The water temperature of the geyser was recorded at 90 °C, and the pH was 7. The litter samples were placed in a plastic bag, kept inside an ice box during transportation to the laboratory and stored at 4 °C until use. Isolation was performed using 1% International *Streptomyces* Project (ISP) medium 1 [25], with 2% gellan gum instead of agar, and incubated at 65 °C. Litter samples were air-dried for a few hours, then aseptically cut into small pieces and placed onto ISP 1 medium solidified with 2% (w/v) gellan gum. Plates were incubated at 65 °C for 3–4 weeks. Growing colonies were picked and purified several times on *Thermus* agar medium. The pure isolate was preserved at 4 °C on *Thermus* agar [13], in 20% (v/v) glycerol suspension at −80 °C, and as a lyophilized culture for long-term preservation.

### Phylogenetic analysis of 16S rRNA gene

Genomic DNA (gDNA) of strain LT1-2-5^T^ was prepared using the method of Yabe *et al*. [26]. The 16S rRNA gene was amplified by PCR with universal eubacterial primers, 27F (5′-AGAGTTTGATCATGGCTCGA-3′; positions 8–27 of the *Escherichia coli* 16S rRNA gene) and 1492R (5′-GGCTACCTTGTTACGACTT-3′; positions 1510–1492) [27]. The 16S rRNA gene sequence of strain LT1-2-5^T^ was compared with available sequences in the GenBank/The European Molecular Biology Laboratory (EMBL)/DNA Data Bank of Japan (DDBJ) database using the Basic Local Alignment Search Tool (blast) homology search [28] and analysed using the EzBioCloud server [29]. Multiple alignments of the 16S rRNA gene sequences and a phylogenetic tree construction were conducted using Molecular Evolutionary Genetics Analysis (mega) version 11 [30]. A phylogenetic tree was constructed with the neighbour-joining (NJ) [31], maximum-likelihood (ML) [32] and minimum-evolution (ME) [33] methods. The evolutionary distances were calculated using Kimura’s two-parameter model [34]. Bootstrap analysis (1,000 replications) was applied to assess the reliability of the tree [35].

**Fig. 1.**
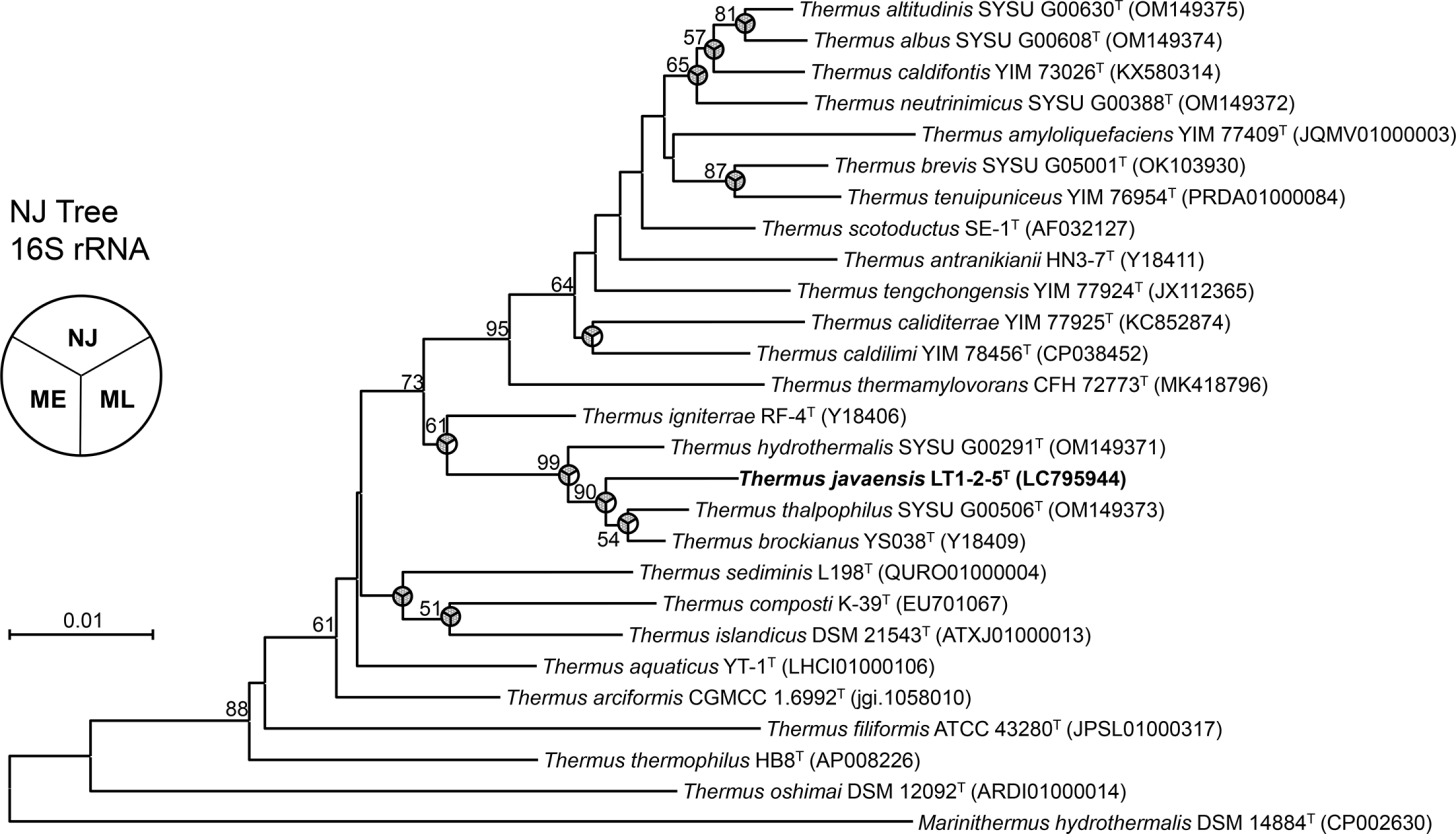
NJ tree based on 16S rRNA gene sequences of strain LT1-2-5^T^ and members of the genus *Thermus*. The tree was also constructed using ML and ME models. The tree was generated based on 1,312 aligned positions of the 16S rRNA gene sequences. Bootstrap values above 50% (based on 1,000 replications) are represented at the branch points. *M. hydrothermalis* DSM 14884^T^ was included as the outgroup. The bar indicates one substitution per 100 nucleotide positions.

The resulting 1,446 bp of full-length sequence of the 16S rRNA gene of LT1-2-5^T^ (GenBank/EMBL/DDBJ under accession number LC795944) was compared against public databases using blast [28] and the EzBioCloud server [29]. Strain LT1-2-5^T^ shared the closest 16S rRNA gene sequence similarities with *Thermus thalpophilus* SYSU G00506^T^ (98.54%), *Thermus brockianus* YS038^T^ (98.34%) and *Thermus hydrothermalis* SYSU G00291^T^ (98.06%). A phylogenetic tree constructed using the NJ, ML and ME methods is shown in Fig. 1. The phylogenetic analysis revealed that strain LT1-2-5^T^ formed a distinct lineage with *T. thalpophilus* SYSU G00506^T^ and *T. brockianus* YS038^T^ (90% bootstrap support), confirming its phylogenetic uniqueness.

### Genome features and phylogenomics

The strain LT1-2-5^T^ was cultured in ISP 1 broth for 3 days at 55 °C. gDNA of strain LT1-2-5^T^ was extracted using the Gentra Puregene Yeast/Bact. Kit (Qiagen) according to the instructions as described by Zheng *et al*. [36]. The TruSeq Nano DNA Library Preparation Kit (Illumina) was used for library preparation, and the whole-genome sequencing was performed using the Illumina NovaSeq 6000 platform. FastQC version 0.11.5 (http://www.bioinformatics.babraham.ac.uk/projects/fastqc) [37] was used to check the quality of raw sequence data from high-throughput sequencing pipelines, and Trimmomatic version 0.36 (http://www.usadellab.org/cms/?page=trimmomatic) was used to remove adapter sequences and low-quality reads. Jellyfish version 2.2.10 (http://www.genome.umd.edu/jellyfish.html) [38] was used to count k-mers in DNA for genome size prediction, genome coverage confirmation and repeat sequence ratio calculation. SPAdes version 3.14.0 (http://cab.spbu.ru/software/spades/) [39] was used for *de novo* assembly. CheckM version 1.1.0 [40] was used to evaluate genome contamination and completeness. Genome annotations were conducted with the DDBJ Fast Annotation and Submission Tool (DFAST) server (https://dfast.ddbj.nig.ac.jp/) [41]. Genome relatedness was assessed with ANI, average amino acid identity (AAI), percentage of conserved proteins (POCP) and Mash distances. Whole-genome ANI was computed with skani [42] version 0.3.0 (skani sketch; skani triangle). AAI was estimated from Double index alignment of next-generation sequencing data (DIAMOND) [43] reciprocal best hits (RBHs) using version 2.1.9 with thresholds of ≥30% identity and ≥50% query/subject coverage; pairwise weighted AAI was the per cent identity averaged across aligned residues (length-weighted across RBH pairs). The POCP [44] between two genomes was computed with DIAMOND [43] version 2.1.9 blastp searches requiring ≥40% identity and ≥50% coverage in each direction, retaining RBH. POCP was calculated as (2×RBH)/(T_A+T_B)×100, where RBH is the number of reciprocal best-hit orthologues, and T_A and T_B are the total predicted proteins in genomes A and B, respectively [44]. Whole-genome distances were obtained with Mash [45] version 3.1 (mash sketch; mash dist) using a k-mer size of *k*=21 and the default sketch size (*s*=1,000). The dDDH value was determined by the Genome-to-Genome Distance Calculator (GGDC) (http://ggdc.dsmz.de/ggdc.php) in the Type (Strain) Genome Server (TYGS) server using formula *d_4_* [46,47].

**Fig. 2.**
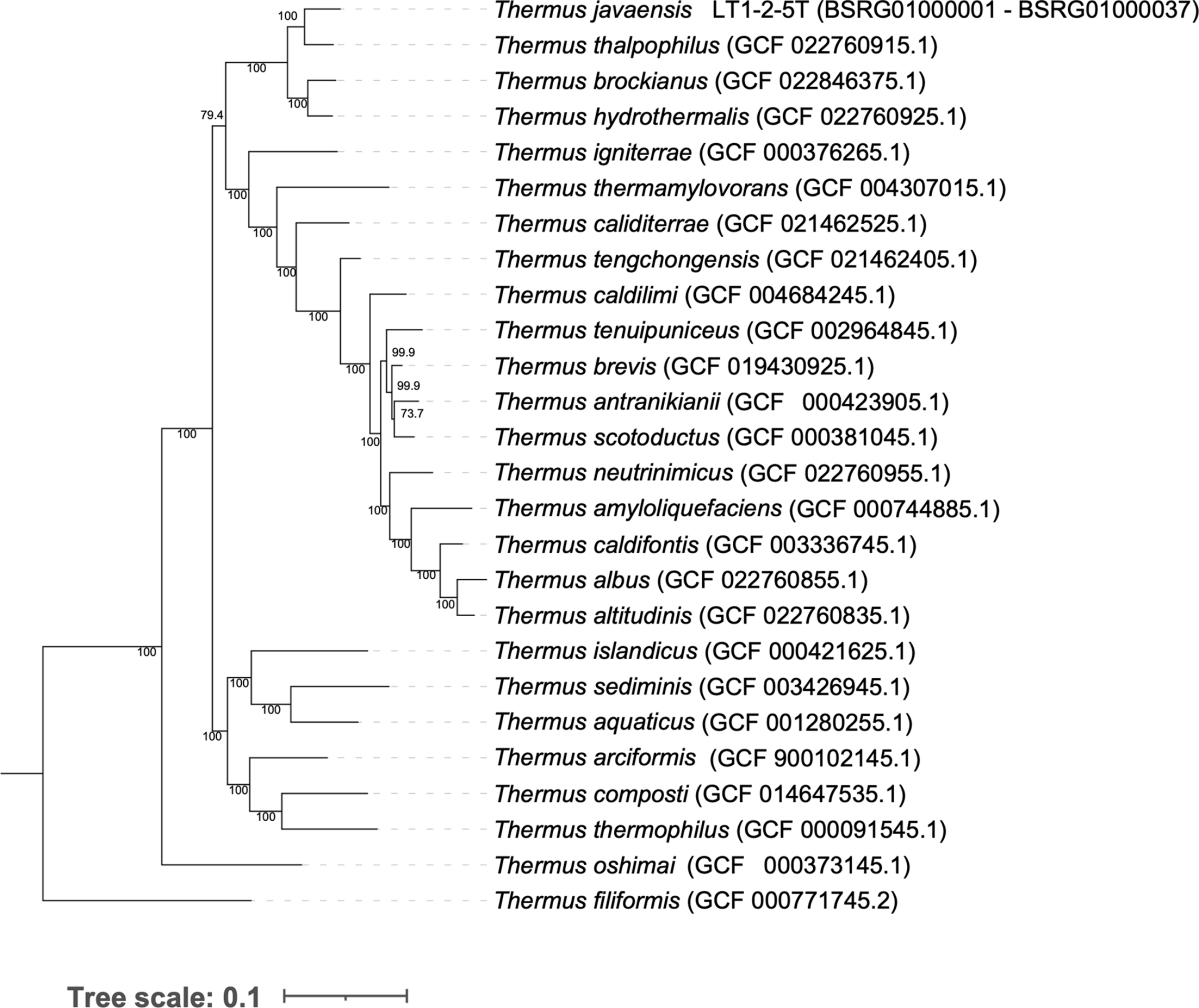
Phylogenomic tree constructed from the whole-genome sequences of strain LT1-2-5^T^ and closely related members of the genus *Thermus*. Genomes were taxonomically screened, GTDB bac120 marker genes were extracted with GTDB-Tk v2.1.1 (GTDB R207), and concatenated amino acid markers were aligned with gtdbtk align (alignment length: 37,288 sites). The alignment was trimmed with trimAl v1.4 using -gt 0.5 -cons 60 and analysed by ML in IQ-TREE v2.3.6 under LG+F+G4 with 1,000 ultrafast bootstraps and 1,000 SH-aLRT tests. The tree was rooted with *M. hydrothermalis* (GCF_000195335.1) and visualized in iTOL. Genome accession numbers are given in parentheses. bac120, a set of 120 conserved single-copy bacterial marker genes; iTOL, interactive tree of life; SH-aLRT, Shimodaira–Hasegawa approximate likelihood ratio test.

The draft genome sequence of strain LT1-2-5^T^ has been deposited at GenBank/EMBL/DDBJ under the genome accession numbers BSRG01000001–BSRG01000037. The genome sequence of strain LT1-2-5^T^ was 2.41 Mbp in size, comprising 37 contigs, with the smallest contig at 50% (*N_50_*) from the total length was being 174,296 bp. Genome completeness and contamination were 99.68% and 1.55%, respectively. The DNA G+C content was 66.78 mol%. A total of 2,516 coding sequences, 3 rRNAs, 48 tRNAs and 8 Clustered Regularly Interspaced Short Palindromic Repeats (CRISPRs) were inferred from the genome of strain LT1-2-5^T^. The ANI values of strain LT1-2-5^T^ compared with its closely related type strains of species of the genus *Thermus* were 89.36–91.46% (Table 1), which were below the 95% cut-off value for species delineation [48]. The highest ANI value of strain LT1-2-5^T^ compared with the type strain *T. thalpophilus* SYSU G00506^T^ was 91.46%. The dDDH values of strain LT1-2-5^T^ compared with its closely related species were 33.9–42.9% (Table 1), which were below the 70% threshold value for species delimitation [46]. The highest dDDH value of strain LT1-2-5^T^ compared with the type strain *T. thalpophilus* SYSU G00506^T^ was 42.9%. The whole-genome-based taxonomic analysis was conducted using a dataset of 27 genome sequences from strain LT1-2-5^T^, all validly published species of *Thermus*, and *Marinithermus hydrothermalis* DSM 14884^T^ was selected as an outgroup. The genome sequences for phylogenomic tree construction were downloaded from the National Center for Biotechnology Information (NCBI) server (https://www.ncbi.nlm.nih.gov/datasets/genome/). Genomes were taxonomically screened using a concatenated alignment of 120 bacterial single-copy marker genes (bac120) generated by Genome Taxonomy Database Toolkit (GTDB-Tk) v2.1.1 [49] (GTDB R207). Furthermore, a concatenated amino- acid markers were aligned with GTDB-Tk align command (alignment length: 37,288 sites). The alignment was trimmed with trimAl [50] version 1.4 using -gt 0.5 -cons 60 and analysed by ML in IQ-TREE [51] version 2.3.6 under LG+F+G4 with 1,000 ultrafast bootstraps replication and 1,000 Shimodaira–Hasegawa approximate likelihood ratio test (SH-aLRT). The tree was rooted with *M. hydrothermalis* (GCF_000195335.1) and visualized in interactive tree of life [52]. The whole-genome-based phylogenetic analysis showed that strain LT1-2-5^T^ formed a separate clade from its closest species, * T. thalpophilus* SYSU G00506^T^ (Fig. 2). The whole-genome-based phylogenetic tree, along with dDDH and ANI values, supported that the strain LT1-2-5^T^ represented a novel species in the genus *Thermus*.

**Table 1.** General features of the genome sequencing and assembly of strain LT1-2-5^T^ and members of the genus *Thermus* Taxa: 1, *T. javaensis* sp. nov. LT1-2-5^T^; 2, *T. thalpophilus* SYSU G00506^T^; 3, *T. brockianus* SNM4-1; 4, *T. hydrothermalis* SYSU G00291^T^.

Characteristic	1	2	3	4
Accession	BSRG01000001–BSRG01000037	GCF_022760915.1	GCF_022846375.1	GCF_022760925.1
G+C content (mol%)	66.78	67.25	66.80	66.95
Genome size (bp)	2,409,297	2,459,001	2,423,923	2,360,711
Scaffolds	37	179	3	38
*N*_*50*_ (bp)	174,296	57,009	2,046,081	114,105
Completeness (%)	99.68	99.65	100	99.68
Contamination (%)	1.55	1.51	0	0.56
No. of CDSs	2,516	2,572	2,531	2,484
No. of rRNA	3	2	6	2
No. of tRNA	48	51	52	49
No. of CRISPRs	8	5	8	4
ANI (%) versus LT1-2-5^T^	–	91.46	89.36	89.51
dDDH (%) versus LT1-2-5^T^	–	42.9	33.9	34.5
AAI (%) versus LT1-2-5^T^	–	91.54	90.07	90.09
POCP (%) versus LT1-2-5^T^	–	81.32	81.07	80.69

The genome length, G+C content and number of CDSs were obtained from the DFAST server (http://www.dfast.nig.ac.jp) [41]. Completeness and contamination scores were computed using CheckM in DFAST. The dDDH values were computed using formula *d_4_* of the GGDC (http://ggdc.dsmz.de/ggdc.php) with the 70% dDDH threshold [42]. The ANI value was calculated using the skani version 0.3.0 (skani sketch; skani triangle) [42].

CDSs, coding sequences; CRISPRs, Clustered Regularly Interspaced Short Palindromic Repeats.

The antiSMASH version 8.0.4 (https://antismash.secondarymetabolites.org) [53] tool was used to predict secondary metabolic biosynthetic gene clusters (SMBGCs) within the genome of strain LT1-2-5^T^ using strictness ‘strict’. This strain harbours only one SMBGC, a terpene, which has no similarity confidence (cluster similarities of <15%) to the known SMBGCs.

### Morphology, physiological and biochemical analyses

**Fig. 3.**
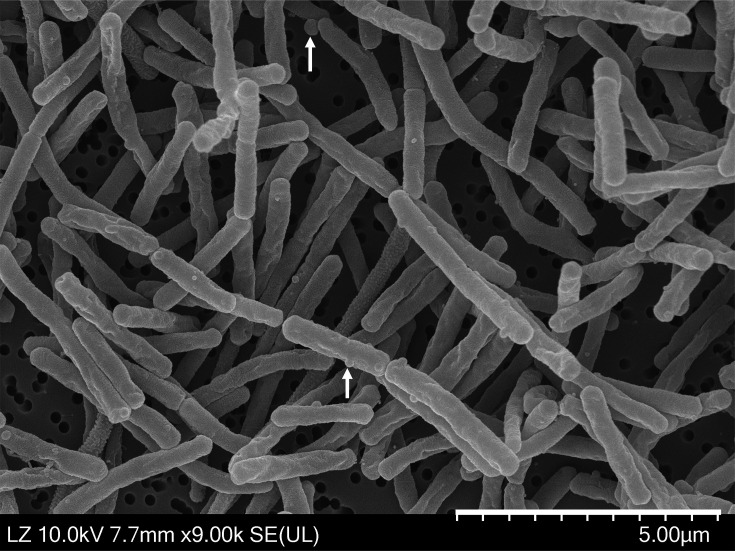
Scanning electron micrograph of strain LT1-2-5^T^. Cells of strain LT1-2-5^T^ were grown on ISP 1 gellan gum at 60 °C for 3 days. Arrows indicate rotund bodies. The SEM image was acquired at 9,000× magnification using a SE detector, with an accelerating voltage of 10.0 kV and a working distance of 7.7 mm. The scale bar = 5.0 µm. SE, secondary electron. LZ, low Z-contrast (material contrast). UL, ultra-low voltage/landing energy.

Colony morphology was observed on cells grown on *Thermus* and ISP 1 agar media at 65 °C for 3 days of incubation. Colonies of strain LT1-2-5^T^ exhibited yellow pigment on both media. Scanning electron microscopy (SEM) preparation for strain LT1-2-5^T^ was performed according to the method described by Le Han *et al*. [54]. Cells were fixed at 4 °C using 2% (w/v) glutaraldehyde in 0.1 M sodium cacodylate buffer, pH 7.4. Cell structures were examined using a Regulus Series FE-SEM (Hitachi). A 3-day-old culture on ISP 1 gellan gum at 60 °C under aerobic conditions formed rod-shaped cells, measuring 0.3–0.5 µm in diameter, with variable length and short filaments. The spherical structures resembling rotund bodies were observed to arise from rod-shaped cells, which were first observed in *T. aquaticus* by Brock and Edwards [55] (Fig. 3).

The growth under anaerobic conditions on *Thermus* agar plates was investigated using an anaerobic chamber (Mitsubishi Gas Chemical) at 65 °C for 1 week. Gram reaction was determined as described by Magee *et al*. [56]. Growth at different temperatures (30, 37, 40, 45, 50, 60, 65, 80 and 82 °C) was examined in 5 ml *Thermus* medium (1% inoculum) in 20 ml tubes with screw cap for 3–7 days. Growth at different pH values was conducted at 65 °C in 200 ml Erlenmeyer flasks containing 50 ml *Thermus* medium (1% inoculum; covered with aluminium foil to prevent evaporation and contamination) in a reciprocal shaker for 3–5 days. The pH range was examined by using 30 mM MES for pH values between 5.0 and 6.5, 30 mM Tris for pH values between 7.0 and 8.5 and 30 mM CAPS for pH values between 9.0 and 11.0. The pH of each buffer was adjusted with hydrochloric acid (HCl) and sodium hydroxide (NaOH) at room temperature. Control media containing each buffer adjusted to pH 8.2 were used to assess the possible inhibitory effects of the buffering agents [3]. Hydrolysis of carboxymethyl cellulose (CMC) (0.1%, w/v), gelatine (0.4%, w/v), l-tyrosine (0.5%, w/v), Tween 20 (1%, w/v), chitin (1%, w/v), casein (1%, w/v) and starch (1%, w/v) was tested on *Thermus* agar media at 65 °C. All experiments were conducted in triplicate. Biochemical characteristics and enzyme activities for strain LT1-2-5^T^ were examined at an incubation temperature of 65 °C for 12 h using API ZYM, and for 24 h using API 20NE and API 50CH systems (bioMérieux) according to the manufacturer’s instructions.

Physiological and biochemical comparison of strain LT1-2-5^T^ with other species in the genus *Thermus* is given in Table 2. Strain LT1-2-5^T^ was Gram-stain-negative, rod-shaped, aerobic bacteria that grew well in *Thermus* and ISP 1 media. Growth was observed at pH and temperatures ranging from pH 6.0 to 10.0 and 45 to 80 °C, respectively. Optimal growth occurred at pH 7.0–8.0 and temperatures of 60–65 °C. Gelatine, l-tyrosine and chitin were hydrolysed. Detection of enzyme activity with API ZYM, 20NE and 50CH strips showed that strain LT1-2-5^T^ had alkaline phosphatase, esterase (C4), esterase lipase (C8), acid phosphatase, naphthol-AS-BI-phosphohydrolase, *α*-glucosidase and *β*-glucosidase, and was able to hydrolyse urea, gelatine, aesculin, d-lyxose, d-tagatose and potassium 5-ketogluconate.

**Table 2.** Phenotypic characteristics of strain LT1-2-5^T^ and related species of the genus *Thermus* Taxa: 1, *T. javaensis* sp. nov. LT1-2-5^T^ (this study); 2, *T. thalpophilus* SYSU G00506^T^ [62]; 3, *T. brockianus* JCM 11602^T^ [3]; 4, *T. hydrothermalis* SYSU G00291^T^ [62]. Strain LT1-2-5^T^ was characterized in this study. Data for strains 2–4 were from the literature [3,62]. API ZYM, API 20NE and API 50CH data of strain JCM 11602^T^ (=NBRC 110747^T^), major fatty acids (>10%) of strains SYSU G00506^T^ (=KCTC 43359^T^), JCM 11602^T^ (=NBRC 110747^T^) and SYSU G00291^T^ (=KCTC 43357^T^) and the major menaquinone of strain SYSU G00291^T^ (=KCTC 43357^T^) were obtained in this study. +, Positive; −, negative; w, weakly positive; nd, no data.

Characteristic	1	2	3	4
**Colour of colony**	**Yellow**	Yellow	Yellow	Yellow
**Temperature range** (°**C**)	**45–80**	50–80	45–75	45–75
**Catalase**	**+**	+	nd	nd
**pH for growth**	**5.5–10**	6–8	5–10	6–8
**Growth in NaCl (%, w/v**)	**0–2**	0–1	0–1.0	0–1.5
**Ability to hydrolyse:**				
Starch	−	nd	−	nd
Casein	−	nd	+	nd
Gelatine	**+**	nd	−	nd
l-Tyrosine	**+**	nd	+	nd
Tween20	−	−	−	+
CMC	−	nd	−	nd
Chitin	**+**	nd	+	nd
**API ZYM:**				
Alkaline phosphatase	**+**	+	**+**	+
Esterase (C4)	**+**	+	−	+
Esterase lipase (C8)	**+**	+	**+**	+
Lipase (C14)	−	+	**+**	−
Leucine arylamidase	−	−	−	+
Valine arylamidase	−	−	−	−
Cysteine arylamidase	−	−	−	−
Trypsin	−	−	−	−
*α*-Chymotrypsin	−	−	−	−
Acid phosphatase	**+**	+	**+**	+
Naphthol-AS-BI-phosphohydrolase	**+**	+	**+**	+
*α*-Galactosidase	−	+	**+**	−
*β*-Galactosidase	−	−	**+**	+
*β*-Glucuronidase	−	−	**+**	−
*α*-Glucosidase	**+**	+	**+**	+
*β*-Glucosidase	**+**	+	**+**	+
*N*-Acetyl-*β*-glucosaminidase	−	−	−	−
*α*-Mannosidase	−	−	−	−
*α*-Fucosidase	−	−	−	−
**API 20NE:**				
Reduction of NO_3_	−	+	**+**	+
l-Tryptophan	−	−	−	nd
d-Glucose	−	−	−	nd
l-Arginine	**+**	−	**+**	nd
Hydrolysis of urea	**+**	−	**+**	nd
Hydrolysis of aesculin	**+**	+	**+**	nd
Hydrolysis of gelatine	**+**	+	**+**	nd
*p*-Nitrophenyl-*β*-d-galactopyranoside	−	+	**+**	nd
d-Glucose	−	+	−	+
l-Arabinose	−	nd	−	nd
d-Mannose	−	+	−	+
d-Mannitol	−	nd	−	nd
*N*-Acetyl-*β*-glucosaminidase	−	nd	−	nd
d-Maltose	−	−	**+**	−
Potassium gluconate	−	nd	−	nd
*n*-Capric acid	−	nd	−	nd
Adipic acid	−	nd	−	nd
dl-Malic acid	−	nd	−	nd
Sodium citrate	−	nd	−	nd
Phenyl acetic acid	−	nd	−	nd
**API 50CH:**				
Aesculin	**+**	nd	**+**	nd
d-Lyxose	**+**	nd	−	nd
d-Tagatose	**+**	nd	**+**	nd
Potassium 5-ketogluconate	**+**	nd	**+**	nd
**Major fatty acids (>10%**)	**Iso-C_17:0_, iso-C_15:0_, anteiso-C_15:0_, anteiso-C_17:0_**	**Iso-C_15:0_, iso-C_17:0_**	**Iso-C_17:0_, iso-C_15:0_**	**Iso-C_17:0_, iso-C_15:0_, anteiso-C_17:0_**
**Polar lipids**	**DPG, PG, PE, GL**	PL, AL, GL	PL, GL	PL, GL
**Menaquinone**	**MK-8**	**MK-8**	**MK-8**	**MK-8**

Tests conducted in this study were indicated in boldface fonts.

AL, aminolipid; NO_3_, nitrate; PL, unidentified phospholipid.

The biochemical characteristics that differentiate strain LT1-2-5^T^ from its closest species, *T. thalpophilus* SYSU G00506^T^, are as follows: strain LT1-2-5^T^ showed no activities of lipase (C14), *α*-galactosidase, nitrate reduction and *p*-nitrophenyl-*β*-d-galactopyranoside, and was not able to utilize d-mannose and d-glucose, but was positive for urea hydrolysis and showed activity of arginine dihydrolase. In contrast, its closest related species, *T. thalpophilus* SYSU G00506^T^, showed positive activities of lipase (C14), *α*-galactosidase, nitrate reduction and *p*-nitrophenyl-*β*-d-galactopyranoside, was able to utilize d-mannose and d-glucose, but was negative for urea hydrolysis and showed no activity of arginine dihydrolase (Table 2).

### Chemotaxonomic analysis

Analysis of cellular fatty acids of LT1-2-5^T^ and reference type strains was performed using cells grown on ISP 1 broth medium at 60 °C with shaking at 100 r.p.m. for 24 h, according to the instructions of the Microbial Identification System (Sherlock TSBA Library version 3.80; Microbial ID, Inc.) [57], and GC (model 6890 N; Agilent Technologies, Palo Alto, CA, USA) using Sherlock MIDI software version 6.2 and the TSBA6 database. Cell walls were prepared using the methods described by Schleifer and Kandler [58]. Polar lipids of strain LT1-2-5^T^ were determined by two-dimensional TLC, as described by Minnikin *et al*. [59,60], using biomass cultured in liquid *Thermus* medium at 65 °C, 100 r.p.m., for 3 days. Analysis for menaquinone was conducted for strains LT1-2-5^T^ and *T. hydrothermalis* SYSU G00291^T^ (=KCTC 43357^T^) using cells grown on ISP 1 broth medium at 60 °C, shaking at 100 r.p.m. for 24 h, as described by Collins *et al*. [61], and identified by HPLC system (Shimadzu Corporation) using a ZORBAX reversed-phase SB-C18 column (Agilent Technologies, Palo Alto, CA, USA).

Chemotaxonomic characteristics of strain LT1-2-5^T^ and closely related species are summarized in Table 2. The major fatty acid profile of strain LT1-2-5^T^ (>10%) comprised iso-C_17:0_ (34.06%), iso-C_15:0_ (24.05%), anteiso-C_15:0_ (13.41%) and anteiso-C_17:0_ (12.79%), consistent with genus-level patterns [19] and those of three other closely related species (Table 3). The major polar lipids of strain LT1-2-5^T^ comprised diphosphatidylglycerol (DPG), phosphatidylglycerol (PG), phosphatidylethanolamine (PE) and two unidentified glycolipids (GLs) (Fig. S1, available in the online Supplementary Material). The major quinone was MK-8.

**Table 3.** Major cellular fatty acids (%) in strain LT1-2-5^T^ and reference species Strains: 1, LT1-2-5^T^; 2, *T. thalpophilus* KCTC 43359^T^ (=SYSU G00506^T^); 3, *T. brockianus* NBRC 110747^T^ (=JCM 11602^T^); 4, *T. hydrothermalis* KCTC 43357^T^ (=SYSU G00291^T^). All data in this table are determined in this study. Cells used in this analysis were grown on ISP 1 broth medium at 60 °C with shaking at 100 r.p.m. for 24 h. Only fatty acids with a content greater than 1.0% are shown. Summed feature 3, C_16:1_ *ω*7*c* and/or *ω*6*c*; summed feature 4, C_17:1_ iso 1 and/or anteiso B; summed feature 5, C_18:2_ *ω*6,9*c* and/or C_18:0_ anteiso; summed feature 8, C_18:1_ *ω*7*c* and/or *ω*6*c*.

Fatty acid	1	2	3	4
**Straight chain**				
C_10:0_	tr	tr	tr	tr
C_14:0_	tr	tr	tr	tr
C_15:0_	–	–	–	–
C_16:0_	6.8	7.1	8.1	7.7
C_17:0_	tr	tr	tr	tr
C_18:0_	2.7	4.8	1.8	3.3
**Unsaturated**				
C_17:1_ iso *ω*10*c*	tr	1.1	tr	–
C_18:3_ *ω*6*c* (6,9,12)	tr	tr	tr	TR
C_18:1_ *ω*9*c*	tr	1.8	tr	tr
**Branched**				
C_11:0_ iso	tr	tr	tr	tr
C_13:0_ iso	tr	3.6	tr	tr
C_14:0_ iso	tr	tr	tr	tr
C_15:0_ iso	24.1	47.7	24.1	14.5
C_15:0_ anteiso	13.4	7.5	4.6	5.1
C_16:0_ iso	1.3	tr	3.9	2.0
C_17:0_ iso	34.1	17.9	46.3	50.8
C_17:0_ anteiso	12.8	2.7	6.2	10.3
C_18:0_ iso	tr	–	tr	tr
C_19:0_ iso	tr	–	tr	TR
**Hydroxy**				
C_15:0_ iso 3-OH	tr	tr	tr	tr
C_15:0_ 2-OH	tr	–	–	–
C_17:0_ iso 3-OH	tr	tr	tr	tr
C_17:0_ 2-OH	tr	–	–	tr
**Summed features**				
Summed feature 3	tr	tr	tr	tr
Summed feature 4	tr	–	–	–
Summed feature 5	tr	tr	tr	tr
Summed feature 8	tr	1.4	tr	tr

2-OH, 2-hydroxy; 3-OH, 3-hydroxy; TR, trace amount <1%.

The chemotaxonomic characteristic that differentiates strain LT1-2-5^T^ from its closest species, *T. thalpophilus* SYSU G00506^T^, was the fatty acids profile. The major fatty acids (>10%) of strain LT1-2-5^T^ were iso-C_17:0_, iso-C_15:0_, anteiso-C_15:0_ and anteiso-C_17:0_, while those of *T. thalpophilus* SYSU G00506^T^ were iso-C_15:0_ and iso-C_17:0_. Meanwhile, differences in polar lipids composition were observed in strain LT1-2-5^T^, which comprised DPG, PG, PE and GLs (Fig. S1), compared to *T. thalpophilus* SYSU G00506^T^, which lacked DPG, PG and PE, as reported by Li *et al*. [62].

Therefore, on the basis of phylogenetic and phylogenomic analyses, as well as phenotypic and chemotaxonomic differences with the most closely related type strains (Tables 1–3), LT1-2-5^T^ is considered a representative of a novel species in the genus *Thermus*, for which the name *T. javaensis* sp. nov. is proposed.

## Description of *Thermus javaensis* sp. nov.

*Thermus javaensis* (ja.va.en’sis. N.L. masc. adj. *javaensis*, refers to Java Island, Indonesia, where the type strain was isolated).

The strain is Gram-stain-negative and aerobic. Cells are 1.8–3.7 µm in length and 0.3–0.5 µm in width. Cells are rod-shaped (0.3–0.5 µm in diameter with variable length) and form short filaments. The spherical structures resembling rotund bodies were observed to arise from rod-shaped cells. Strain showed yellow-pigmented colonies on *Thermus* and ISP 1 media after 3 days of incubation. It grows at 45–80 °C (optimum 60–65 °C), at pH 6.0–10.0 (optimum pH 7.0–8.0) and with 0–2% (w/v) sodium chloride (NaCl). Positive enzyme activities include alkaline phosphatase, esterase (C4), esterase lipase (C8), acid phosphatase, naphthol-AS-BI-phosphohydrolase, *α*-glucosidase and *β*-glucosidase; negative enzyme activities include lipase (C14), leucine arylamidase, valine arylamidase, cystine arylamidase, trypsin, *α*-chymotrypsin, *α*-galactosidase, *β*-galactosidase, *β*-glucuronidase, *N*-acetyl-*β*-galactosaminidase, *α*-mannosidase and *α*-fucosidase (API ZYM system). Positive for hydrolysis of l-arginine, urea, aesculin and gelatine in the API 20NE system. Positive for hydrolysis of d-lyxose, d-tagatose and potassium 5-ketogluconate in the API 50CH system. Positive for catalase activity. Hydrolyses l-tyrosine and chitin, but not starch, casein, Tween 20 or CMC. The major menaquinone is MK-8. The major polar lipids are DPG, PG, PE and GLs. The major fatty acids (>10%) of strain LT1-2-5^T^ comprised iso-C_17:0_, iso-C_15:0_, anteiso-C_15:0_ and anteiso-C_17:0_. The genome size and G+C content are 2.41 Mbp and 66.8 mol%, respectively.

The type strain is LT1-2-5^T^ (=UICC B-84^T^=CGMCC 1.61921^T^=KCTC 102310^T^), isolated from litter around a geyser in Cisolok, West Java, Indonesia. The draft whole-genome sequences of the type strain LT1-2-5^T^ have been deposited in DDBJ under the accession numbers BSRG01000001–BSRG01000037, and the GenBank accession number for the 16S rRNA gene sequence of the type strain LT1-2-5^T^ is LC795944.

## Supplementary material

10.1099/ijsem.0.007136 Fig. S1.

## Abbreviations

AAI: average amino acid identity
AL: aminolipid
ANI: average nucleotide identity
blast: Basic Local Alignment Search Tool
CDS: coding sequences
CMC: carboxymethyl cellulose
CRISPRs: Clustered Regularly Interspaced Short Palindromic Repeats
DDBJ: DNA Data Bank of Japan
dDDH: digital DNA–DNA hybridization
DFAST: DDBJ Fast Annotation and Submission Tool
DIAMOND: double index alignment of next-generation sequencing data
DPG: diphosphatidylglycerol
EMBL: The European Molecular Biology Laboratory
gDNA: genomic DNA
GGDC: Genome-to-Genome Distance Calculator
GL: unidentified glycolipid
GTDB: Genome Taxonomy Database
HCl: Hydrochloric acid
ISP: International *Streptomyces* Project
iTOL: interactive tree of life
LPSN: List of Prokaryotic Names with Standing in Nomenclature
ME: minimum-evolution
mega: Molecular Evolutionary Genetics Analysis
ML: maximum-likelihood
NaCl: sodium chloride
NaOH: sodium hydroxide
NCBI: National Center for Biotechnology Information
NJ: neighbour-joining
NO3: nitrate
PE: phosphatidylethanolamine
PG: phosphatidylglycerol
PL: unidentified phospholipid
POCP: percentage of conserved proteins
RBHs: reciprocal best hits
SE: secondary electron
SEM: Scanning electron microscopy
SH-aLRT: Shimodaira–Hasegawa approximate likelihood ratio test
SMBGCs: secondary metabolic biosynthetic gene clusters
TR: trace
TYGS: Type (Strain) Genome Server
