# Supplementary material for: Thermus javaensis sp. nov., a novel thermophilic bacterium isolated from litter of a geyser in Cisolok, West Java, Indonesia
Source: Int J Syst Evol Microbiol. 2026 May 4;76(5):007136. doi: 10.1099/ijsem.0.007136 (PMC13142892; doi:10.1099/ijsem.0.007136)
Supplement: Fig. S1. [file ijsem-76-07136-s001.pdf]

Supplementary Data

Thermus javaensis, sp. nov., a novel thermophilic bacterium isolated from litter of a geyser in Cisolok, West Java, Indonesia

Fitria Ningsih[1,2], Mazytha Kinanti Rachmania[1,2], Dhian Chitra Ayu Fitria Sari[2], Yasunori Ichihashi[3], Song-Gun Kim[4], Wellyzar Sjamsuridzal[1,2*], Shuhei Yabe [3,5,6*]

[1]Department of Biology, Faculty of Mathematics and Natural Sciences, Universitas Indonesia, Kampus UI Depok, 16424, Indonesia

[2]Center of Excellence for Indigenous Biological Resources-Genome Studies, Faculty of Mathematics and Natural Sciences, Universitas Indonesia, Kampus UI Depok, 16424, Indonesia

[3]RIKEN Center for Sustainable Resource Science, 3-1-1 Koyadai, Tsukuba, Ibaraki, 305-0074, Japan

[4]Biological Resource Center / Korean Collection for Type Cultures (KCTC), Korea Research Institute of Bioscience and Biotechnology, Jeongeup, Jeonbuk 56212, Republic of Korea

[5]Department of Microbial Resources, Graduate School of Agricultural Science, Faculty of Agriculture, Tohoku University, 468-1 Aramaki Aza Aoba, Aoba-ku, Sendai, Miyagi, 980-8572, Japan

[6]Hazaka Plant Research Center, Kennan Eisei Kogyo Co.Ltd., 44 Inariyama, Ashitate, Shibata-gun, Miyagi, 989-1311, Japan

*** Corresponding authors:**

1. Shuhei Yabe, shuhei.yabe@riken.jp

2. Wellyzar Sjamsuridzal, sjwelly@sci.ui.ac.id

Supplementary data contains one figure.

Legend to the figure in the supplementary material

Fig S1.

The polar lipid profile of strain LT1-2-5^T. Chromatograms were developed in the first dimension with chloroform-methanol-water (65:25:4, v/v) and in the second with chloroform-acetic acid-methanol-water (85:15:12:4), v/v). Spraying with 10% ethanolic molybdophosporic acid and heated at 160°C for 15–20 min.

Abbreviations: DPG: Diphosphatidylglycerol; PG: Phosphatidylglycerol; PE: Phosphatidylethanolamine; GL: unknown glycolipid.

Supplementary Figure S1.

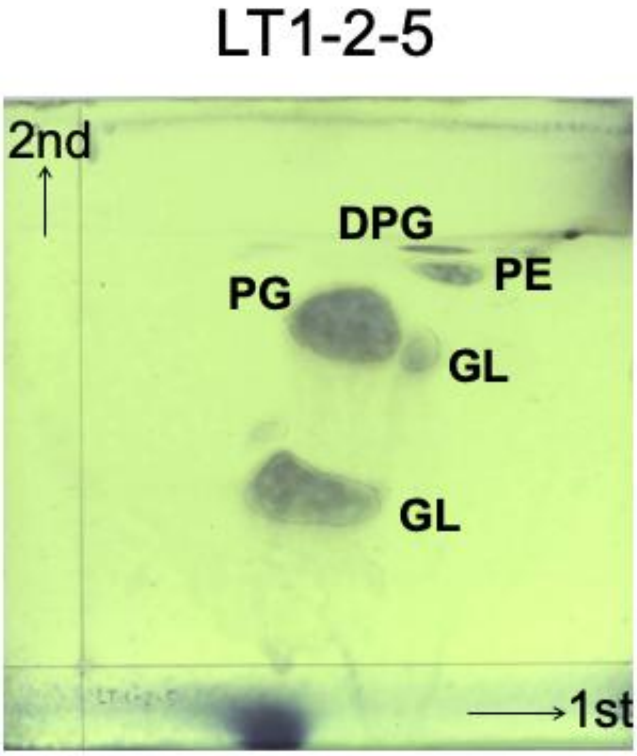

Fig S1.

The polar lipid profile of strain LT1-2-5^{T}. Chromatograms were developed in the first dimension with chloroform-methanol-water (65:25:4, v/v) and in the second with chloroform-acetic acid-methanol-water (85:15:12:4), v/v). Spraying with 10% ethanolic molybdophosporic acid and heated at 160°C for 15–20 min.

Abbreviations: DPG: Diphosphatidylglycerol; PG: Phosphatidylglycerol; PE: Phosphatidylethanolamine; GL: unknown glycolipid.
